# Environmental impacts of mass drug administration programs: exposures, risks, and mitigation of antimicrobial resistance

**DOI:** 10.1186/s40249-022-01000-z

**Published:** 2022-06-30

**Authors:** Joanna K. Konopka, Pranab Chatterjee, Connor LaMontagne, Joe Brown

**Affiliations:** 1 The Solomon H. Snyder Department of Neuroscience, Johns Hopkins University School of Medicine, Baltimore, MD 21205 USA; 2 Department of International Health, Johns Hopkins Bloomberg School of Public Health, Johns Hopkins University, Baltimore, MD 21205 USA; 3 Department of Environmental Sciences and Engineering, Gillings School of Global Public Health, University of North Carolina at Chapel Hill, Chapel Hill, NC 27599-7431 USA

**Keywords:** Antibiotics, Mass drug administration, Environment, Antimicrobial resistance

## Abstract

Mass drug administration (MDA) of antimicrobials has shown promise in the reduction and potential elimination of a variety of neglected tropical diseases (NTDs). However, with antimicrobial resistance (AMR) becoming a global crisis, the risks posed by widespread antimicrobial use need to be evaluated. As the role of the environment in AMR emergence and dissemination has become increasingly recognized, it is likewise crucial to establish the role of MDA in environmental AMR pollution, along with the potential impacts of such pollution. This review presents the current state of knowledge on the antimicrobial compounds, resistant organisms, and antimicrobial resistance genes in MDA trials, routes of these determinants into the environment, and their persistence and ecological impacts, particularly in low and middle-income countries where these trials are most common. From the few studies directly evaluating AMR outcomes in azithromycin MDA trials, it is becoming apparent that MDA efforts can increase carriage and excretion of resistant pathogens in a lasting way. However, research on these outcomes for other antimicrobials used in MDA trials is sorely needed. Furthermore, while paths of AMR determinants from human waste to the environment and their persistence thereafter are supported by the literature, quantitative information on the scope and likelihood of this is largely absent. We recommend some mitigative approaches that would be valuable to consider in future MDA efforts. This review stands to be a valuable resource for researchers and policymakers seeking to evaluate the impacts of MDA.

**Graphical Abstract:**

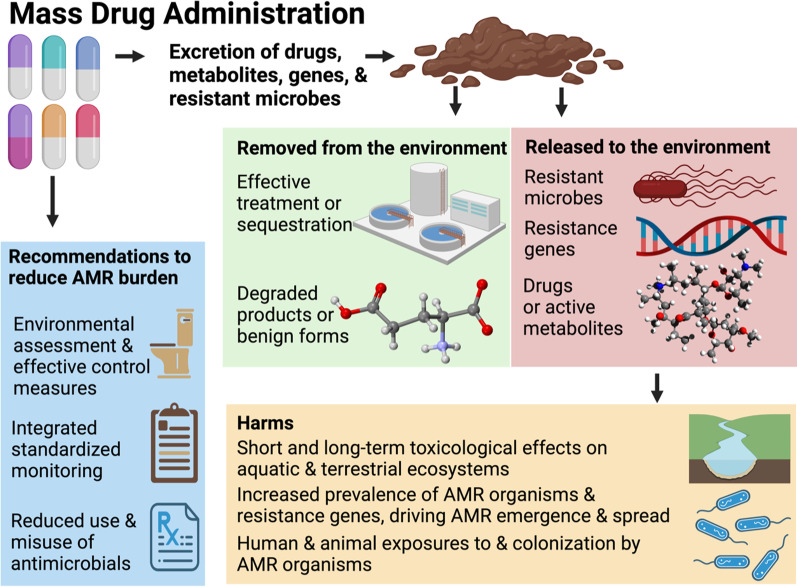

## Background

Mass drug administration (MDA) programs have been successfully used as both prevention and treatment of neglected tropical diseases (NTDs), especially in low- and middle-income countries (LMICs) [1]. While the success of MDA programs might contribute to reducing childhood morbidity and mortality related to NTDs at the population level [2–5]. Potentially adverse environmental effects of those programs must be carefully weighed alongside expected benefits and adverse events of MDA in humans [6, 7]. A hallmark of MDA programs is to eliminate the need for individual diagnosis and to administer treatment to all members in a given community at the same time, regardless of clinical presentation. This dramatically expanded use of antimicrobial agents (either individually or in combination) in a small geographic area, over a short time (weeks to months), can result in high quantities of unmetabolized drugs and bioactive compounds being introduced into the environment [8]. With most MDA programs using antimicrobial agents (including antibiotics and antiparasitics) [9], it is important to consider how the frequency of administration of those agents, and the doses in which they are administered, might be contributing to antimicrobial resistance (AMR) in the environment. Yet, environmental monitoring to assess AMR in the aftermath of MDA programs has been largely neglected.

AMR is the ability of microorganisms to withstand or resist the action of one or more antimicrobial agents, making them no longer responsive to drug treatments meant to eliminate them. At the population level, antimicrobial (e.g., antibiotic) administration can select for emergence and spread of such resistant strains. Environmental compartments experiencing strong anthropogenic pressures are particularly susceptible to antimicrobial contamination and thus are at high risk of increased AMR [10, 11]. This resistance can be acquired through mutations or exchange of fragments or entire resistance genes via horizontal gene transfer (HGT) in the microbial community [12].

The exacerbation of AMR in the environment is multifactorial in nature and is thus difficult to assess and measure [13, 14]. The emergence and spread of AMR are at least partly caused by the human–environment interactions and the intensification of human activities which demand increased antibiotic use. These human activities include: (i) intensification in agriculture, fisheries, and crop production to meet the global challenges to sustain food production and meat/fish-rich diets; (ii) intensification of mass movements associated with urbanization, trade, and travel, as well as mass human and non-human animal migrations, and (iii) industrialization and land use changes, leading to increased pollution favoring pathogen transmission.

Additionally, the impact of antimicrobial environmental contamination will not be the same everywhere and requires case-by-case analysis and risk assessment. Different LMICs can be affected by specific local problems as well as regional issues, leading to different outcomes. Among the environmental factors that determine risks of antibiotic contaminants are the composition of the microbial community as well as water and nutrient amounts already present in the environment (e.g., from manure) [15]. These factors, in turn, determine how quickly the populations of resistant microbes can grow and exchange resistance genes with other members of the microbial community, thus affecting transmission and persistence of AMR in the environment. Additional determinants that need to be taken into consideration are human population size and overcrowding, state of and access to water-sanitation-hygiene infrastructure, presence and seasonality of enteric infections, as well as location, amount, and periodicity of antibiotic discharge (e.g. single vs repeated) in a given community.

The risk of contaminating the environment with antimicrobial residues, particularly in the aftermath of MDA activities, raises concerns about the environmental threat of AMR [16]. These scenarios remain especially likely in settings with inadequate waste containment and treatment [17]. Micro- and macro-organisms (e.g., phyto- and zooplankton, fish, insects) in contact with such contaminated water and soil are at risk of being exposed to those compounds, which can lead to unanticipated impacts on their survival and fitness. Additionally, those exposed organisms (including but not limited to those in the microbial community) can acquire antibiotic resistance genes (ARGs) selected for via MDA. Combined, these risks call for closer examination of the emergence of AMR or transmission of ARGs to humans and other animals via the environment. If MDA plays a substantial role in AMR spread, the short-term health benefits may be outweighed by long-term loss of antimicrobial efficacy.

In this review, we synthesize the current state of knowledge on introduction of antimicrobials and their metabolites into the environment in the context of MDA and discuss MDA’s potential impact on ecosystems in resource-limited settings. The most commonly used antibacterial agent in MDA programs is the macrolide antibiotic azithromycin for treatment, prevention, and control of trachoma and yaws [18–20]. We predominantly outline the role of azithromycin in generation of AMR and ARGs, and discuss other antimicrobial agents where data exist. We trace these compounds from the time they are excreted into the environment from humans and discuss the exposures, risks, and opportunities for mitigation of AMR.

## Methods

Data for this scoping review were initially identified through a search of PubMed, Web of Science, and Google Scholar, using the keywords “mass drug administration,” “antibiotic,” and “ecology OR eco*.” This search (conducted throughout the month of October 2021) provided an initial set of studies relevant to the topic. References from the initially identified studies were also reviewed and included if relevant. Only articles published in English were included. The authors did not use exclusion criteria based on year of publication and added further references based on their knowledge of the subject area. A consultation with a panel of subject matter experts was conducted prior to manuscript submission to evaluate the accuracy and completeness of this review. These experts have been selected for their expertise in key areas being addressed by this review, including environmental microbiology, antimicrobial resistance, and environmental health. Specifically, these experts specialize in civil and environmental engineering; environmental microbiology and chemistry; environmental AMR emergence and dissemination; water, sanitation, and hygiene; and agricultural AMR. These experts are not authors of this paper and their names, affiliations, and positions have been listed in the acknowledgements section.

## MDA and AMR

### Prevalence of AMR in humans following MDA programs

The available evidence suggests that azithromycin MDA can increase the prevalence of AMR across human communities in a widespread and lasting way [21–25]. In these studies, azithromycin-resistant *Escherichia coli* and *Streptococcus pneumoniae* were detected via rectal or nasopharyngeal swabs in as many as 55–68% of individuals in treated communities. This is compared to much lower (often single digit) prevalence in baseline and control communities [25]. While these studies focused on selected pathogens, antibiotics often act on a wide range of microbes, and therefore resistance can develop even in harmless bystander organisms living in humans. These microbes are often missed when AMR surveillance focuses on specific organisms and not ARGs in general. Genetic determinants of macrolide resistance studied in the Niger arm of the MORDOR trial (semi-annual azithromycin MDA) were elevated 7.5 times by the 3-year and 4-year timepoints [26]. Although prevalence of resistance did decline with time, elevated AMR levels were maintained in the populations for as long as 6 months, even with just a single MDA dose [22, 23]. With no monitoring beyond the timeline of those studies, it is possible that AMR levels remained elevated for longer. Furthermore, even when azithromycin is administered on its own, it can increase rates of resistance to other macrolides like erythromycin as well as to clinically relevant non-macrolide antibiotics like trimethoprim/sulfamethoxazole, beta-lactams, and aminoglycosides [24, 27]. This co-selection of resistance to antibiotics other than those being administered is well-recognized, and may occur via a variety of mechanisms, including genes for different resistances being located on the same mobile genetic element (and therefore passed as a unit between microbes) and one ARG being useful for resisting multiple types of antibiotics [28]. The Cochrane Review of Trachoma control echoes the above assessment of the literature as supporting a risk of lasting multidrug AMR development in response to azithromycin MDA [29]. In total, prolonged elevated levels of AMR in patients may defeat the original purpose of the MDA effort by introducing more persistent infections and thus less treatable disease, as noted elsewhere [30].

Literature on impacts of MDA on resistance to other antimicrobials is sparse. Resistance is a concern for MDA campaigns against schistosomiasis, as it relies on a single drug, praziquantel [31]. AMR in soil-transmitted helminths (STH) is expected to develop slowly from MDA. Yet, the availability of only two benzimidazole antihelminth drugs (albendazole and mebendazole) and limited development of novel drugs are problematic should antihelminthic resistance arise [32]. Decreased efficacy of these benzimidazoles over the past two decades has already been reported [33]. Although there is a lack of direct evidence connecting lowered antihelminthic drug efficacy in humans and MDA for control of STHs [34], livestock animals may serve as a proxy for examination of resistance trends in response to MDA. Livestock often receive regular doses of the antimicrobials used in human MDA. Intestinal nematodes resisting benzimidazoles and ivermectin are already a serious problem in livestock globally and these trends are expected to continue as parasites are exposed to selective pressure [9, 35].

Resistance to the antimalarial artemisinin has become widespread in the Greater Mekong Subregion in Southeast Asia [36], and antimalarial MDA is considered to encourage resistance [37, 38]. However, it has been suggested that well-managed MDA may not give rise to AMR in humans [39–41]. Proper management involves use of artemisinin-based combination treatment (ACT), reaching high coverage and adherence, timing implementation when transmission is lowest, and strong surveillance [41]. A similar combination strategy might be beneficial in MDA programs using antibiotics to reduce the rise of AMR, provided that special care is taken to reduce bystander selection.

## Environmental introduction of AMR

Once established in a human (e.g., colonization of the gut), drug resistant pathogens and resistance genes may spread into the environment via human waste. Gut carriage of resistant pathogens or ARGs may be prevalent even in healthy individuals [42] and elevated in LMIC settings [43], and these organisms may be selected for in individuals receiving antibiotics. Of particular concern in LMIC settings where large MDA trials are becoming more common is the potential role of inadequate sanitation in accelerating the emergence and dissemination of AMR in the environment [44]. These issues have not been extensively explored in settings where antibiotic stewardship and sanitation infrastructure are both poor [45], despite the likely role of AMR hotspots to lead to drug resistant phenotypes that may threaten global public health [46–48]. AMR can also be found in waste disposal systems such as pit latrines (fecal sludges and aqueous effluent from decentralized sanitation) and wastewater treatment plants (wastewater, biosolids), where improper containment or inadequate treatment may then allow spread through runoff, seepage, or direct flow into groundwater, surface water, or other discharges to the environment [49, 50]. ARGs and associated resistant phenotypes can thus spread rapidly within and between different communities as contaminated waste is mobilized [51]. Multi-drug resistant enteric pathogens, such as carbapenem-resistant Enterobacteriaceae, are currently highlighted among the top global concerns in AMR [52]. A recent global burden of disease assessment of deaths attributable to AMR bacteria estimated that *E. coli*, *Klebsiella pneumoniae*, and *Pseudomonas aeruginosa* were among the six most common pathogens directly responsible for AMR-associated deaths globally, each causing more than 250,000 deaths in 2019 [53]. These three bacteria are enriched in feces, and the feces-associated *E. coli* bacterium was the top such resistant pathogen, causing an estimated 23.4% (95% UI: 19.5–28.2) of total global deaths attributable to AMR and 24.3% (95% UI: 22.9–25.8) of total deaths associated with AMR in 2019 [53]. Inadequate sanitation results in the release of up to an estimated 6.5 × 10^10^ kg of feces globally containing extended-spectrum beta lactamase (ESBL) producing *E. coli*, a figure that is expected to double by 2030 [54].

Antimicrobial resistant organisms are not the only AMR determinants found in waste from individuals receiving these drugs. Antimicrobials themselves may not be completely absorbed or metabolized by the body and are excreted in feces and urine, though this varies significantly by drug. For instance, substantial amounts of azithromycin are excreted unchanged (around 21%), while albendazole and its pharmacologically active metabolite albendazole sulfoxide are only excreted in minute amounts [55, 56]. Non-antibiotic antimicrobials seem to be absorbed more completely in general, though more pharmacokinetic research on these drugs would be valuable [57–59]. Once excreted, these compounds may follow the same paths as resistant organisms into the environment. For instance, upon entering wastewater systems, such antimicrobial residues have been described to follow one of three fates: biodegradation [60, 61], adsorption into sludge [62, 63], or emergence unchanged as pharmaceutical pollutants in the aquatic environment [64, 65]. The fate, transport, and persistence of antibiotic residues and metabolites in environmental media are not well understood. Studies from agricultural runoff suggest that such compounds may be present at elevated concentrations for weeks [66], and ARGs may remain elevated for longer. Persistence is likely to be a function of a range of biotic and abiotic environmental conditions and may be specific to the antibiotic. Of course, antibiotic loading to the environment in highly impacted settings may result in long-term release of these compounds and their metabolites over time, allowing concentrations in environmental media to remain elevated or accumulate.

Thus, human waste in LMICs may introduce antimicrobials and other determinants of AMR into the environment. The degree and timescale of such introduction will depend on drug excretion rates, waste management infrastructure, and the kinetics of persistence in the environment. Environmental introduction of AMR resulting from MDA can be expected to continue for as long as elevated AMR levels were observed in the MDA-AMR studies highlighted above (e.g., at least 6 months). Indeed, AMR spread to environments in and around the home (i.e., the more immediate “environment” such as household soil) are also recognized [67], in particular as a locus of potentially high exposure. Domestic environments in LMICs may receive high levels of fecal loading both from non-human animals and inadequate sanitation at the household level [68–70].

### Environmental risks, resistance determinants, and persistence

The persistence of resistant pathogens and other AMR determinants depends on several factors. Once in the environment, resistant pathogens vary in their ability to survive. For many pathogens, environmental conditions are too far removed from those inside human hosts for sustained survival. For more adaptable organisms like *E. coli* [71], soils and other environmental media represent important potential reservoirs, allowing for spread and persistence of ARGs [72]. *Escherichia coli* and related taxa have been observed as naturalized in both tropical and even temperate climates [71]. Even if a resistant organism cannot survive for long in its new surroundings, it may pass its ARGs to more suitable microbes by horizontal gene transfer (HGT) via the processes of transformation, transduction, or conjugation [73, 74]. ARGs have been shown to persist in environmental settings across time [75], and, in the case of water environments, across geographies [76, 77].

Though these processes are clearly occurring, research evaluating their likelihood in environmental settings is largely absent. Such research would aid in establishing risks posed by releases of ARGs, antibiotics and their residues or metabolites, and AMR organisms into the environment. Despite the occurrence of these microbial processes, ARGs traditionally have been expected to be gradually lost from environmental organisms. This is because ARGs might not provide enough benefit to outweigh their fitness costs in the absence of exposure to therapeutic concentrations of antimicrobials [78]. However, even concentrations of antibiotics hundreds of times below a bacterium’s minimum inhibitory concentration (i.e., the threshold known to inhibit bacterial growth) have been shown to select for AMR under controlled laboratory settings [79, 80]. This suggests that antimicrobial compounds excreted by MDA participants might increase the persistence of ARGs in the environment, even when diffuse. These studies were conducted in controlled laboratory settings, so research in the environment (settings with variations in heat, UV exposure and salinity) would be needed to better support the validity of this phenomenon. MDA in single-dose regimens (e.g., 20 mg/kg azithromycin) may represent lower overall concentrations than typical therapeutic doses (administered over several days) [81] and possibly lower than in agricultural applications where dosing may be highly variable [82]. This will certainly vary by compound, as different drugs display different rates of degradation in the environment [83]. The degree to which non-antibiotic antimicrobial resistance genes persist in the environment is not known, though it may be possible.

### Ecotoxicology

The presence of pharmaceutical agents in the environment can lead to emergence of AMR or selection for resistance, as well as non-target effects on other organisms as a result of direct (i.e., physical contact with the pharmaceutical agents or their metabolites) or indirect (i.e., secondary contact with other affected organisms or individuals) exposures [84]. The specific role of MDA programs in causing such environmental contamination with antimicrobial agents is largely unknown. The emergence of AMR is influenced by a wide range of drivers that are prevalent in settings where MDA is gaining currency. While there are growing concerns of the deleterious effect of chronic, low-grade exposure to human pharmaceutical agents in the environment, targeted studies exploring the ecological impacts of specific agents remain sparse [85].

The largest body of evidence of non-target effects of pharmaceuticals in the environment exists for antiparasitic or anti-helminthic drugs such as ivermectin. In addition to being used in agricultural context for farm animals, ivermectin is frequently employed in MDA programs to control diseases such as onchocerciasis and filariasis in humans. Most of the human and non-human administered ivermectin is eliminated in the feces [58, 86]. A wide range of lethal and sublethal effects on soil communities, especially invertebrate fauna, have been recorded in response to ivermectin (Table 1). The insecticidal action of excreted ivermectin is responsible for reduced biodiversity [87, 88] or elimination [89] of dung beetles. Even in low doses, ivermectin can significantly impair locomotion and ability to reproduce in beneficial cow-dung insect communities. Such a reduction in invertebrate activity can in turn affect important ecosystem services they provide, including dung degradation, soil fertilization and seed dispersal [89, 90].

**Table 1:** Non-target environmental effects of pharmaceuticals used in MDA programs

Pharmaceutical agent	Organism	Concentrations/amount used	Reported effects	Type of experiment	Additional study characteristics	Timescale	Refs.
Ivermectin (antiparasitic)	Dung beetles (insects)	100 μg/kg of ivermectin	Reduced species richness, abundance, and biomass	Field (Spain)	Spiked dung exposed in the field	2 seasons (spring and autumn) Sampling 12 and 48 h after dung placement	[87]
		40 µg/kg/day	Reduced dung degradation associated with absence of dung-degrading insects	Field (England)	Faeces of calves fitted with rumenal boluses delivering ivermectin	100 days	[89]
		1.0, 3.3, 10.0, 33.3, 100.0 and 200.0 μg/kg	Impaired locomotion, reduced foraging success, death	Laboratory	Non-contaminated bovine dung from ivermectin-free cattle	12−18 days	[91]
	Dung insect community (Diptera and Hymenoptera)	6.6 µg/kg fresh dung	Reduced biodiversity	Field (Switzerland)	Fresh cattle dung collected on the local farms	24 cattle farms; repeated over 3 seasons	[88]
Azithromycin (antibiotic)	Algae (phytoplankton)	0.5 and 1 μg/L (low) 5–100 μg/L (high)	Accelerated growth (low dose) Inhibited growth and disrupted photosynthesis (high dose)	Laboratory	Algae grown in 1 L flasks	96 h	[94]
		LC50	Growth inhibition	Laboratory	13 antibiotics tested Algae in the exponential growth phase exposed to antibiotics 250 ml conical flasks used	96 h	[95]
	Daphnia (zooplankton)	1, 10, 50, 100 and 200 mg/L	Altered feeding behavior and nutrition accumulation	Laboratory	Different exposure pathways investigated (aqueous phase and food phase)	Up to 96 h	[96]
	European sea bass (fish)	0.625, 1.25, 2.5, 5, 10 and 20 mg/L	Larval mortality and morphological abnormalities (at 20 mg/L)	Laboratory	Sea bass obtained from aquaculture, kept in 1 L aquaria with seawater	96 h for acute toxicity 4 and 14 days for chronic toxicity	[97]
	Zebrafish (fish)	10 and 50 μg/L	Cardiotoxicity	Laboratory	Macrolide antibiotics dissolved in embryos medium	Up to 5 days post fertilization of fish embryos	[98]
	Tilapia (fish)	1, 50 and 100 mg/L	Considered non-toxic (with moderate liver damage)	Laboratory	Fish obtained from aquaculture 3 fish/aquarium; 1 g fish/L	48 h (acute toxicity) 14 days (chronic exposure)	[99]
	Marine fish	N/A (field sample collection)	Bioaccumulation in livers	Field (China)	7 wild fish species collected from Laizhou Bay, North China using bottom trawl Tissues dissected and tested for antibiotics	One time collection	[100]
	Oyster, mussel, and clam (bivalve molluscs)	Field-sample collection	Bioaccumulation	Field and Laboratory (Spain)	Molluscs field-collected Homogenized tissues tested	2 collections 1 month apart	[101]
	Earthworms	0.0089 to 0.03 mg/kg and 0.16 mg/kg	No toxicity response Bioaccumulation of antibiotic in the tissues with potential of entry into food webs	Laboratory	1 L Mason jars filled with soil Uses both environmentally relevant and unrealistically high concentrations	28 days	[102]
	Radish, lettuce, and fescue grass (plants)	0.83 and 3.2 mg/kg	Minimal toxicity and bioaccumulation	Laboratory	Biosolids-amended soils and soils directly spiked with antibiotics	32 days after planting (radish); 46 days after planting (lettuce); 42 days after planting (fescue grass)	[103]
	Microbial community	Low, medium, and high (based on Targeted National Sewage Sludge Survey	Minimal toxicity	Laboratory	Direct application of biosolids or antibiotic mixture to soil 300 mL glass jars with soil	120 days	[102]
Other mixtures	Microbial community	0.1 or 10 mg/kg soil of antibiotic mixture containing azithromycin	Increase the abundance of novel antibiotic resistance genes identified via functional metagenomics in the soil	Field (Canada)	Annual application Quadruplicate plots	8 years	[104]
	Microbial community	75 dry t/ha of compost	Increase in gene targets for macrolide resistance, persisting in soil for up to 4 years	Field (Canada)	One time application of compost 4 blocks of 5 plots (12.2 m wide by 10 m long)	10 years	[105]

*MDA* mass drug administration, *N/A* not applicable

Less is known about potential ecotoxicological effects of antibiotics released in the environment. In humans, nearly half of an oral dose of azithromycin is excreted unchanged in stool, and about 6% in urine [55, 91–93]. Thus, as a result of MDA programs, a large quantity of this antibiotic is likely to be discharged into the environment. Although no evidence of the ecotoxicological impact of azithromycin is available in the context of MDA programs, laboratory studies suggest that its presence in the water or soil can negatively affect some organisms (e.g., phyto- and zooplankton) [94–96].

The majority of negative effects of azithromycin are recorded in aquatic ecosystems (Table 1). Azithromycin accelerates algal growth and disrupts algal photosynthesis at low and high doses, respectively [94]. In fact, of 13 tested antibiotics, azithromycin was the most toxic one to freshwater green-algae based on calculated EC50 values (i.e., concentrations of the compounds causing 50% algal growth inhibition compared to control) over a 96 h period [95]. Azithromycin also alters feeding behavior and nutrition accumulation of the zooplankton *Daphnia magna* [96]. Since algae and zooplankton form the basis of aquatic food webs, drastic and sudden changes in their population growth (as could be the case following MDA in settings with insufficient sanitation infrastructure) would likely cause a rippling effect through the entire ecosystem, especially in small bodies of water (e.g., creeks, ponds, drainage ditches etc.) with limited resilience. At higher trophic levels, azithromycin can cause larval morality, morphological abnormalities, and cardiotoxicity in European sea bass and zebrafish [97, 98]. It causes moderate liver damage but is considered non-toxic in tilapia [99]. In addition to the lethal and sublethal effects, azithromycin has high bioaccumulation capacity in fish, and was detected in tissues of several fish and bivalves [100, 101]. Thus, when not lethal, the accumulation of this antibiotic in animal tissues would ensure its presence in the ecosystem and possible movement through the food web, including back to humans via regular and frequent consumption of such animal tissues.

Similarly, little is known about the effects of azithromycin on terrestrial ecosystems (Table 1). Most of the antibiotics in soils are introduced via the application of biosolids or livestock manure. Minimal toxicity was reported for microbial communities following direct application of biosolids or antibiotic mixture (including azithromycin) to soils at realistic doses in a short-term (up to 120 days) microcosm setup [102]. Similarly, at environmentally relevant concentrations, there was minimal toxicity and accumulation in food plants including radish and lettuce [103]. While no toxicity response was noted for earthworms in azithromycin-treated soil, they accumulated the antibiotic in their tissues [102]. On the other hand, long-term field studies indicate that at high concentrations and with repeated application, antibiotic mixture containing azithromycin can increase the abundance of ARGs (novel antibiotic resistance genes identified via functional metagenomics) in the soil [104]. Furthermore, one-time application of compost from various sources (including swine manure as well as food and yard waste) led to an increase in gene targets for macrolide resistance, which persisted in the soil for up to 4 years [105]. Thus, while the nutrient function and availability in soil would likely be unaffected in response to azithromycin, this macrolide has the potential to bioaccumulate, enter the food web, and increase the abundance of ARGs in soil over time (Table 1).

Despite the lack of studies directly evaluating the impact of MDA programs on environmental levels of antimicrobials and their subsequent ecological toxicity, there is cause for concern. Such effects are not easily anticipated and can depend on type and length of exposure in the environment. The persistence of azithromycin and ARG targets in the environment as well as bioaccumulation in some animal tissues calls for more careful monitoring in the context of MDA programs. Given a wide range of toxicological effects in aquatic systems, many of which are sublethal (i.e., do not kill the organism), environmental assessments should consider more than just biodiversity (i.e., the number or presence of various organisms in the ecosystem) and look for sublethal effects as early warning signs. When large human populations are dosed simultaneously with an antibiotic during MDA programs, even modest excretions could cause significant and long lasting ecotoxicological effects. Environmental risk assessment exercises and long-term monitoring conducted in conjunction with MDA programs could provide insights into the unintended impacts of the use of antimicrobials simultaneously on a large scale.

### Environmental AMR exposures and human health

The human health risks posed by environmental AMR are not well understood, and direct evidence of AMR transmission from the environment to humans is lacking. A growing body of microbiological literature is showing extensive shared resistance between humans, non-human animals, and the environment, particularly in LMIC settings [106, 107]. However, this research does not discern whether the direction of AMR sharing is from humans to the environment, vice-versa, or both. One study has been able to suggest transmission from the environment to humans—using an in-depth, multi-method approach, Yoon et al. argued that an amikacin resistance gene travelled from the environmental species *Acinetobacter guillouiae* to the highly problematic clinical pathogen *Acinetobacter baumanii* [108]. Aside from this, the bulk of work in this area focuses on theoretical frameworks for assessing human health risks of environmental AMR, which point to risk factors such as presence of an ARG on a mobile genetic element or presence within a human pathogen [109, 110]. Quantitative, data-driven research is still needed to develop our understanding of this potential pathway for AMR dissemination.

Ultimately, the risk of exposure to environmental AMR is likely to be heterogenous, with higher risk to communities living in areas with greater antimicrobial agent exposure and poor water, sanitation, and hygiene (WASH) infrastructure or environmental protection measures. Indeed, multiple potential routes of exposure to resistant pathogens in low-resource settings are recognized, such as washing foods with contaminated water, floods, and children playing in open drains and cesspools [44]. Rapid urbanization and population growth further exacerbate factors promoting the risk of AMR transmission from environmental sources [111]. Thus, there are likely differences in how AMR spreads in rural communities (e.g., less likely to possess adequate WASH infrastructure) versus urban communities (e.g., more crowding) in LMICs. However, it is not clear whether these differences position rural or urban communities as more susceptible to environmental AMR dissemination and reinfection.

## Strategies for mitigation of AMR following MDA

While the risks are so far poorly characterized, mitigation strategies are needed to effectively limit potentially serious adverse impacts of AMR linked to MDA programming. Modeling in the context of malaria elimination MDA programs indicates a wide range of factors may be associated with the emergence of drug resistance [39]. Identifying which factors are associated with AMR in the context of MDA programs is complicated by the role of co-selection of resistance genes or alterations in gut microbiomes, which may augment the sharing of ARGs or emergence of AMR organisms.

One approach to mitigate the environmental risk of AMR is to treat wastewater effluent to remove antimicrobial pollutants. There is no direct data on how MDA programs affect environmental antimicrobial pollution. However, hospital wastewater contaminates environmental compartments with AMR organisms or ARGs. Hospital effluents have higher concentrations of AMR organisms and ARGs, and they have a diverse group of AMR determinants, often enriched by fecal contamination [76, 112–116]. Although treatment of such effluents using conventional wastewater management strategies may reduce the burden of AMR organisms [117], variation exists in the amounts of antimicrobial residues that can be removed by wastewater treatment processes [118]. Removing antimicrobial residues from urban wastewater is a difficult process, and this could be an even larger problem in the setting of MDA programs in LMICs [119]. An estimated 3.6 billion people (approximately 46% of the global population) lacked safely managed sanitation in 2020 [120]. Without sanitation infrastructure that can properly contain human excreta, downstream exposures to wastes containing antibiotics, metabolites, ARGs, and AR pathogens is possible. Treatment of waste streams to remove AMR determinants would be even more challenging in these circumstances.

Use of antimicrobials has become a “quick fix” for deeply entrenched problems in many LMICs [121]. Mitigation strategies need to be identified for the short- and long-term control of adverse impacts of MDA on antimicrobial residues, AMR organisms and ARGs in the environment. Access to and consistent use of appropriate WASH infrastructure, both at the community level and in healthcare facilities, could reduce the transmission of AMR organisms. It also has the added benefit of reducing the spread of infectious diseases in general, thus limiting the need for MDA [122]. The relatively low coverage of WASH infrastructure in healthcare facilities in LMICs further increases the chances of emergent AMR infections in such settings, especially in an environment with high levels of antimicrobial pollution [123]. Targeted hygiene services, supply of clean potable water, and access to appropriate sanitation facilities have been associated with lower levels of circulating AMR organisms in community settings and in healthcare facilities [124].

Longitudinal environmental impact assessments should accompany ongoing or future MDA efforts. With multiple MDA programs and trials on the horizon, there is an opportunity to capitalize on ongoing efforts to address key knowledge gaps about how MDA affects environmental compartments [125]. In addition, innovative options, such as wastewater surveillance, provide synergies between pathogen detection [126] and AMR containment efforts. Wastewater monitoring could be used to examine not only the impact of MDA programs on environmental antimicrobial pollution, but also their impact on emergence of AMR organisms and spread of ARGs. Such a monitoring system would provide early signals of emergent AMR threats, and background data against which the impact of MDA programs and AMR mitigation efforts can be assessed. Existing wastewater surveillance systems for polio or COVID-19 can provide a starting point [127, 128] possibly building on the World Health Organization Tricycle Protocol [129] that focuses on ESBL *E. coli* monitoring in environmental matrices [130]. This protocol has been designed specifically for cost-effective, at-scale AMR monitoring in LMICs.

Risk assessment efforts should guide decision-making for using azithromycin MDA to improve child survival. Modeling approaches could be employed to identify countries or communities which are likely to have the most benefit from MDA [131]. These frameworks, in combination with an environmental surveillance system, can be used to construct an early warning system for MDA-associated AMR. Some experts have also recommended using a multi-criteria decision tool, in consultation with the relevant local stakeholders, prior to expanding the use of azithromycin as an agent for MDA [5].

## Recommendations

While this review has identified and assessed the available evidence of the potential environmental impacts of MDA, many research questions remain (Table 2). Here, we provide the following recommendations for stakeholders, including policy makers:

- Integrated, standardized monitoring of AMR emergence and dissemination alongside MDA programs using appropriate designs and measures. At minimum, trials should incorporate pre- and post-intervention monitoring with appropriate controls across time scales that will capture proximal and medium-term effects of MDA on phenotypic and genotypic resistance measures in microbes of interest.
- Monitoring concentrations of MDA-relevant antimicrobials in waste streams and environments around treated populations. These concentrations would be compared to concentrations expected to promote resistance in environmental organisms, such as predicted no effect concentrations (PNECs) [132], in order to evaluate the ongoing threat of AMR emergence.
- Environmental impact assessments of ecotoxicological effects on organisms in the environment should consider not only population sizes and biodiversity but also include measures of sublethal effects as early warning signs.
- Case-by-case risk assessment analysis of the impacts of antimicrobial environmental contamination in different communities to account for the multifactorial nature of the AMR exacerbation. Special care is needed to account for local and regional variations (both environmental and human in nature).
- Modeling of known and predicted risk factors to determine which interventions could have the greatest impact.

**Table 2:** Evidence assessment and the identified research gaps regarding the antimicrobial resistance (AMR) in the environment in the context of mass drug administration (MDA) programs

Research area	Strength of evidence	Remaining gaps
AMR produced by MDA	Moderate	AMR impacts of MDA with antimicrobials other than azithromycin
Environmental introduction of AMR and antimicrobials	Strong	The role of inadequate sanitation in AMR emergence in settings with poor antibiotic stewardship and sanitation infrastructure Pharmacokinetics of non-antibiotic antimicrobials in MDA studies Persistence of antimicrobials in environmental settings
Environmental persistence of AMR	Moderate	The likelihood of AMR dissemination via HGT in environmental settings Differences between ARGs and genes for resistance to other antimicrobials
Ecotoxicology	Limited	Ecotoxicological effects of MDA-relevant antimicrobials other than azithromycin and ivermectin Ecotoxicological effects in response to MDA
Transmission of AMR from the environment to humans	Limited	Quantitative understanding of the risk posed by environmental ARGs

*AMR* antimicrobial resistance, *HGT* horizontal gene transfer, *ARG* antibiotic resistance genes

## Conclusions

This review identified the growing evidence base around the environmental threats associated with MDA programs. Despite there being very few studies conducting environmental risk assessments alongside MDA programs, there are causes for concern. When administered on a population level, antimicrobials which may be discharged through urine and stool, can pose risks including contamination of the environment across scales, and setting off cascades of unintended consequences. Even at low concentrations, these pharmaceuticals can disrupt the balance of ecosystems, especially aquatic ones.

Given the uncertain mechanism behind the improved child survival associated with MDA of azithromycin, it is possible that in the absence of other systemic changes, longer term or multiple MDA efforts may be needed to maintain the successes. While the risk of emergent AMR organisms in short term efforts may be offset by morbidity and mortality prevention, longer term benefits may be attenuated by increasing health risks imposed by emergent AMR.

Multiple MDA programs are being planned with no apparent monitoring to assess the environmental risks and consequences. While these programs might have reduced morbidity and mortality linked to NTDs, it is always preferable to institute systemic changes which can address deeper factors impeding improvement of maternal and child survival. Until then, we suggest that MDA-based approaches should be deployed in combination with environmental risk assessment and mitigation frameworks, to ensure that the ecotoxicological impacts of the excreted drugs are minimized.

## Data Availability

Not applicable.

## Abbreviations

ACT: Artemisinin-based combination treatment
ARG: Antibiotic resistance genes
AMR: Antimicrobial resistance
COVID-19: Coronavirus disease 2019
HGT: Horizontal gene transfer
LMIC: Low-to-middle-income countries
MDA: Mass drug administration
MORDOR: Mortality reduction after oral azithromycin
NTD: Neglected tropical diseases
PNEC: Predicted no effect concentration
STH: Soil-transmitted helminths
WASH: Water, sanitation, and hygiene
